# 3,9-Di-1-naphthyl-2,4,8,10-tetra­oxa­spiro­[5.5]undeca­ne

**DOI:** 10.1107/S1600536810014741

**Published:** 2010-04-28

**Authors:** Xiuqin Zhang, Yuan Cui, Bin Yu, Xiaoqiang Sun, Qiang Chen

**Affiliations:** a High Technology Research Institute of Nanjing University, Changzhou 213162, People’s Republic of China; b Key Laboratory of Fine Petrochemical Engineering, Changzhou University, Changzhou 213162, People’s Republic of China

## Abstract

In the title compound, C_27_H_24_O_4_, the 1,3-dioxane rings have chair conformations. The mol­ecule has non-crystallographic twofold rotation symmetry. The dihedral angle between the naphthalene ring systems is 17.96(4)° In the crystal structure, weak inter­molecular C—H⋯π inter­actions contribute to the crystal packing.

## Related literature

For a related 3,9-diphenyl structure, see: Wang *et al.* (2006 ▶). For other oxaspiro structures, see: Mihis *et al.* (2008 ▶); Shi *et al.* (2009 ▶).
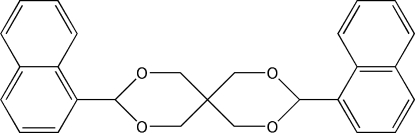


## Experimental

### 

#### Crystal data

C_27_H_24_O_4_
*M* *_r_* = 412.46
Monoclinic, 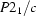

*a* = 14.9040 (15) Å
*b* = 5.7761 (6) Å
*c* = 24.238 (2) Å
β = 95.447 (2)°
*V* = 2077.1 (4) Å^3^
*Z* = 4
Mo *K*α radiation
μ = 0.09 mm^−1^
*T* = 295 K
0.22 × 0.21 × 0.19 mm

#### Data collection

Bruker APEXII CCD diffractometer
Absorption correction: multi-scan (*SADABS*; Bruker, 2003 ▶) *T*_min_ = 0.981, *T*_max_ = 0.984
11731 measured reflections
4067 independent reflections
2961 reflections with *I* > 2σ(*I*)
*R*_int_ = 0.024

#### Refinement

*R*[*F* ^2^ > 2σ(*F* ^2^)] = 0.042
*wR*(*F* ^2^) = 0.158
*S* = 1.03
4067 reflections
280 parameters
H-atom parameters constrained
Δρ_max_ = 0.16 e Å^−3^
Δρ_min_ = −0.17 e Å^−3^

### 

Data collection: *APEX2* (Bruker, 2003 ▶); cell refinement: *SAINT* (Bruker, 2003 ▶); data reduction: *SAINT*; program(s) used to solve structure: *SHELXS97* (Sheldrick, 2008 ▶); program(s) used to refine structure: *SHELXL97* (Sheldrick, 2008 ▶); molecular graphics: *SHELXTL* (Sheldrick, 2008 ▶); software used to prepare material for publication: *SHELXL97* and *PLATON* (Spek, 2009 ▶).

## Supplementary Material

Crystal structure: contains datablocks I, global. DOI: 10.1107/S1600536810014741/si2258sup1.cif

Structure factors: contains datablocks I. DOI: 10.1107/S1600536810014741/si2258Isup2.hkl

Additional supplementary materials:  crystallographic information; 3D view; checkCIF report

## Figures and Tables

**Table 1:** Hydrogen-bond geometry (Å, °) *Cg*5 and *Cg*6 are the centroids of the C18–C23 and C22–C27 rings, respectively.

*D*—H⋯*A*	*D*—H	H⋯*A*	*D*⋯*A*	*D*—H⋯*A*
C16—H16*B*⋯*Cg*5^i^	0.97	2.95	3.5827 (19)	124
C27—H27⋯*Cg*6^ii^	0.93	2.94	3.754 (2)	147

Symmetry codes: (i) 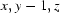; (ii) 
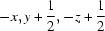
.

**Fig. 1.**
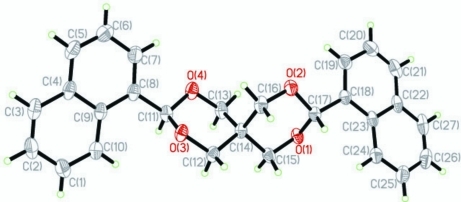
Molecular structure of the title compound with the atom labelling scheme. Displacement ellipsoids are drawn at the 30% probability level and H atoms are drawn as small spheres of arbitrary radii.

## supplementary crystallographic information

### Comment

The title compound is an important intermediate in the synthesis of pesticides. Several related structures were synthesized and reported (Wang *et al.*,2006; Mihis *et al.*,2008; Shi *et al.*,2009). The X-ray structural analysis confirmed the assignment of the structure of the title compound from spectroscopic data. The molecular structure is depicted in Fig. 1. The title molecule has a non-crystallographic twofold rotation symmetry. In the molecules, the naphthalene planes make a dihedral angle of 17.96 (4) °. Weak intermolecular C–H···π interactions contribute to the crystal packing (Table 1).

### Experimental

Pentaerythritol (0.22 g, 1.6 mmol), α-naphthaldehyde (0.5 g, 3.2 mmol), *p*-toluene sulphonic acid (0.02 g, 0.12 mmol) and dimethylbenzene (10 ml) were heated for six hours. The mixture was cooled and then filtered. The organic phase was evaporated on a rotary evaporator and the resulting solid was recrystallized in ethyl acetate, yielding the title compound (0.53 g, 80%); m.p. 445-446 K.

### Refinement

All H atoms were fixed geometrically and treated as riding with C—H=0.93 Å, 0.97 Å or 0.98 Å, and *U*_iso_(H)=1.2*U*_eq_(C-methylene, C-aromatic).

### Crystal data

**Table:**

C_27_H_24_O_4_	*F*(000) = 872
*M**_r_* = 412.46	*D*_x_ = 1.319 Mg m^−^^3^
Monoclinic, *P*2_1_/*c*	Mo *K*α radiation, λ = 0.71073 Å
*a* = 14.9040 (15) Å	Cell parameters from 4101 reflections
*b* = 5.7761 (6) Å	θ = 2.8–28.9°
*c* = 24.238 (2) Å	µ = 0.09 mm^−^^1^
β = 95.447 (2)°	*T* = 295 K
*V* = 2077.1 (4) Å^3^	Block, colorless
*Z* = 4	0.22 × 0.21 × 0.19 mm

### Data collection

**Table:**

Bruker APEXII CCD diffractometer	4067 independent reflections
Radiation source: fine-focus sealed tube	2961 reflections with *I* > 2σ(*I*)
graphite	*R*_int_ = 0.024
φ and ω scans	θ_max_ = 26.0°, θ_min_ = 1.4°
Absorption correction: multi-scan (*SADABS*; Bruker, 2003)	*h* = −18→16
*T*_min_ = 0.981, *T*_max_ = 0.984	*k* = −6→7
11731 measured reflections	*l* = −29→29

### Refinement

**Table:**

Refinement on *F*^2^	Primary atom site location: structure-invariant direct methods
Least-squares matrix: full	Secondary atom site location: difference Fourier map
*R*[*F*^2^ > 2σ(*F*^2^)] = 0.042	Hydrogen site location: inferred from neighbouring sites
*wR*(*F*^2^) = 0.158	H-atom parameters constrained
*S* = 1.03	*w* = 1/[σ^2^(*F*_o_^2^) + (0.1*P*)^2^ + 0.1334*P*] where *P* = (*F*_o_^2^ + 2*F*_c_^2^)/3
4067 reflections	(Δ/σ)_max_ = 0.001
280 parameters	Δρ_max_ = 0.16 e Å^−^^3^
0 restraints	Δρ_min_ = −0.17 e Å^−^^3^

### Special details

**Table:**

Geometry. All esds (except the esd in the dihedral angle between two l.s. planes) are estimated using the full covariance matrix. The cell esds are taken into account individually in the estimation of esds in distances, angles and torsion angles; correlations between esds in cell parameters are only used when they are defined by crystal symmetry. An approximate (isotropic) treatment of cell esds is used for estimating esds involving l.s. planes.
Refinement. Refinement of *F*^2^ against ALL reflections. The weighted *R*-factor wR and goodness of fit *S* are based on *F*^2^, conventional *R*-factors *R* are based on *F*, with *F* set to zero for negative *F*^2^. The threshold expression of *F*^2^ > σ(*F*^2^) is used only for calculating *R*-factors(gt) etc. and is not relevant to the choice of reflections for refinement. *R*-factors based on *F*^2^ are statistically about twice as large as those based on *F*, and *R*- factors based on ALL data will be even larger.

### Fractional atomic coordinates and isotropic or equivalent isotropic displacement parameters (Å^2^)

**Table:**

	*x*	*y*	*z*	*U*_iso_*/*U*_eq_	
O1	0.13414 (8)	0.84377 (18)	0.45655 (4)	0.0446 (3)	
O2	0.22638 (8)	0.6064 (2)	0.40974 (4)	0.0515 (3)	
O3	0.26584 (7)	0.18521 (18)	0.56082 (5)	0.0480 (3)	
O4	0.36401 (8)	0.4833 (2)	0.54471 (5)	0.0547 (3)	
C1	0.34069 (15)	−0.1745 (4)	0.71697 (8)	0.0678 (6)	
H1	0.2942	−0.1732	0.7400	0.081*	
C2	0.40805 (16)	−0.3418 (4)	0.72465 (9)	0.0720 (6)	
H2	0.4063	−0.4513	0.7527	0.086*	
C3	0.47631 (14)	−0.3452 (3)	0.69128 (9)	0.0650 (6)	
H3	0.5207	−0.4584	0.6965	0.078*	
C4	0.48080 (12)	−0.1802 (3)	0.64906 (8)	0.0539 (5)	
C5	0.55262 (13)	−0.1798 (4)	0.61468 (9)	0.0700 (6)	
H5	0.5969	−0.2934	0.6193	0.084*	
C6	0.55731 (14)	−0.0168 (4)	0.57552 (10)	0.0790 (7)	
H6	0.6062	−0.0149	0.5543	0.095*	
C7	0.48923 (13)	0.1507 (4)	0.56622 (9)	0.0653 (5)	
H7	0.4933	0.2611	0.5386	0.078*	
C8	0.41737 (12)	0.1540 (3)	0.59693 (7)	0.0488 (4)	
C9	0.41222 (11)	−0.0103 (3)	0.64054 (7)	0.0463 (4)	
C10	0.34223 (12)	−0.0132 (3)	0.67623 (7)	0.0556 (5)	
H10	0.2966	0.0970	0.6717	0.067*	
C11	0.34087 (11)	0.3197 (3)	0.58327 (7)	0.0460 (4)	
H11	0.3259	0.3980	0.6171	0.055*	
C12	0.18859 (11)	0.3248 (3)	0.54602 (7)	0.0488 (4)	
H12A	0.1679	0.3917	0.5793	0.059*	
H12B	0.1406	0.2283	0.5288	0.059*	
C13	0.29204 (12)	0.6434 (3)	0.53143 (7)	0.0523 (5)	
H13A	0.3104	0.7575	0.5054	0.063*	
H13B	0.2784	0.7239	0.5648	0.063*	
C14	0.20848 (10)	0.5180 (3)	0.50633 (6)	0.0406 (4)	
C15	0.12684 (12)	0.6778 (3)	0.49917 (7)	0.0495 (4)	
H15A	0.0729	0.5857	0.4904	0.059*	
H15B	0.1207	0.7576	0.5338	0.059*	
C16	0.22281 (12)	0.4224 (3)	0.44935 (7)	0.0506 (4)	
H16A	0.2787	0.3353	0.4515	0.061*	
H16B	0.1739	0.3179	0.4373	0.061*	
C17	0.14617 (11)	0.7325 (3)	0.40554 (6)	0.0413 (4)	
H17	0.0954	0.6281	0.3956	0.050*	
C18	0.14747 (11)	0.9196 (3)	0.36260 (6)	0.0430 (4)	
C19	0.22421 (13)	0.9714 (3)	0.33883 (7)	0.0554 (5)	
H19	0.2755	0.8813	0.3470	0.066*	
C20	0.22692 (15)	1.1598 (4)	0.30208 (8)	0.0674 (6)	
H20	0.2796	1.1916	0.2858	0.081*	
C21	0.15360 (16)	1.2942 (4)	0.29038 (7)	0.0670 (6)	
H21	0.1569	1.4206	0.2669	0.080*	
C22	0.07234 (14)	1.2462 (3)	0.31316 (7)	0.0535 (5)	
C23	0.06793 (12)	1.0532 (3)	0.34917 (6)	0.0442 (4)	
C24	−0.01615 (12)	1.0029 (3)	0.36927 (7)	0.0543 (5)	
H24	−0.0207	0.8775	0.3929	0.065*	
C25	−0.09045 (14)	1.1344 (4)	0.35467 (8)	0.0687 (6)	
H25	−0.1452	1.0951	0.3676	0.082*	
C26	−0.08524 (17)	1.3280 (4)	0.32048 (9)	0.0761 (7)	
H26	−0.1359	1.4193	0.3114	0.091*	
C27	−0.00558 (17)	1.3820 (4)	0.30050 (8)	0.0711 (6)	
H27	−0.0024	1.5114	0.2780	0.085*	

### Atomic displacement parameters (Å^2^)

**Table:**

	*U*^11^	*U*^22^	*U*^33^	*U*^12^	*U*^13^	*U*^23^
O1	0.0659 (7)	0.0329 (6)	0.0345 (6)	0.0103 (5)	0.0018 (5)	0.0004 (4)
O2	0.0625 (8)	0.0500 (7)	0.0416 (6)	0.0150 (6)	0.0034 (5)	−0.0012 (5)
O3	0.0504 (7)	0.0305 (6)	0.0603 (7)	0.0003 (5)	−0.0103 (5)	0.0055 (5)
O4	0.0552 (7)	0.0417 (7)	0.0639 (8)	−0.0075 (5)	−0.0122 (6)	0.0112 (6)
C1	0.0774 (14)	0.0711 (14)	0.0534 (11)	−0.0021 (11)	−0.0019 (9)	0.0115 (10)
C2	0.0816 (15)	0.0645 (14)	0.0651 (12)	−0.0064 (11)	−0.0180 (11)	0.0232 (10)
C3	0.0643 (12)	0.0498 (11)	0.0750 (13)	−0.0001 (9)	−0.0245 (10)	0.0110 (9)
C4	0.0495 (10)	0.0471 (10)	0.0603 (10)	−0.0024 (8)	−0.0204 (8)	0.0018 (8)
C5	0.0489 (11)	0.0699 (14)	0.0879 (15)	0.0110 (10)	−0.0104 (10)	0.0089 (12)
C6	0.0557 (12)	0.0890 (17)	0.0927 (16)	0.0088 (12)	0.0089 (11)	0.0187 (14)
C7	0.0582 (12)	0.0679 (13)	0.0684 (12)	−0.0001 (10)	−0.0016 (9)	0.0152 (10)
C8	0.0509 (10)	0.0427 (9)	0.0494 (9)	−0.0029 (8)	−0.0131 (7)	−0.0005 (7)
C9	0.0490 (9)	0.0402 (9)	0.0458 (9)	−0.0050 (7)	−0.0161 (7)	−0.0032 (7)
C10	0.0614 (11)	0.0527 (11)	0.0502 (10)	0.0036 (9)	−0.0078 (8)	0.0022 (8)
C11	0.0553 (10)	0.0358 (9)	0.0443 (8)	−0.0043 (7)	−0.0088 (7)	−0.0008 (7)
C12	0.0504 (10)	0.0360 (9)	0.0587 (10)	0.0043 (7)	−0.0019 (8)	0.0086 (7)
C13	0.0690 (12)	0.0320 (9)	0.0525 (10)	−0.0035 (8)	−0.0110 (8)	0.0037 (7)
C14	0.0500 (9)	0.0295 (8)	0.0410 (8)	0.0029 (7)	−0.0025 (7)	−0.0009 (6)
C15	0.0640 (11)	0.0422 (9)	0.0428 (9)	0.0111 (8)	0.0075 (8)	0.0072 (7)
C16	0.0658 (11)	0.0363 (9)	0.0476 (9)	0.0127 (8)	−0.0055 (8)	−0.0054 (7)
C17	0.0498 (9)	0.0358 (8)	0.0369 (8)	0.0040 (7)	−0.0028 (6)	−0.0062 (6)
C18	0.0547 (10)	0.0423 (9)	0.0307 (7)	−0.0015 (8)	−0.0035 (6)	−0.0065 (7)
C19	0.0613 (11)	0.0614 (12)	0.0429 (9)	−0.0007 (9)	0.0020 (8)	−0.0036 (8)
C20	0.0767 (14)	0.0766 (15)	0.0497 (11)	−0.0168 (12)	0.0099 (10)	0.0028 (10)
C21	0.1016 (17)	0.0567 (12)	0.0406 (9)	−0.0183 (12)	−0.0041 (10)	0.0068 (8)
C22	0.0810 (13)	0.0434 (9)	0.0326 (8)	0.0004 (9)	−0.0125 (8)	−0.0030 (7)
C23	0.0622 (10)	0.0398 (9)	0.0283 (7)	0.0006 (8)	−0.0074 (7)	−0.0060 (6)
C24	0.0612 (11)	0.0571 (11)	0.0429 (9)	0.0068 (9)	−0.0034 (8)	−0.0002 (8)
C25	0.0664 (13)	0.0836 (15)	0.0534 (11)	0.0179 (11)	−0.0090 (9)	−0.0104 (10)
C26	0.0882 (16)	0.0758 (16)	0.0589 (12)	0.0347 (13)	−0.0216 (11)	−0.0084 (11)
C27	0.1127 (19)	0.0499 (11)	0.0449 (10)	0.0161 (12)	−0.0234 (11)	0.0012 (9)

### Geometric parameters (Å, °)

**Table:**

O1—C17	1.4198 (18)	C12—H12B	0.9700
O1—C15	1.4211 (19)	C13—C14	1.517 (2)
O2—C17	1.3954 (18)	C13—H13A	0.9700
O2—C16	1.437 (2)	C13—H13B	0.9700
O3—C12	1.4233 (18)	C14—C16	1.521 (2)
O3—C11	1.4265 (19)	C14—C15	1.524 (2)
O4—C11	1.395 (2)	C15—H15A	0.9700
O4—C13	1.429 (2)	C15—H15B	0.9700
C1—C10	1.360 (3)	C16—H16A	0.9700
C1—C2	1.393 (3)	C16—H16B	0.9700
C1—H1	0.9300	C17—C18	1.502 (2)
C2—C3	1.359 (3)	C17—H17	0.9800
C2—H2	0.9300	C18—C19	1.362 (2)
C3—C4	1.404 (3)	C18—C23	1.426 (2)
C3—H3	0.9300	C19—C20	1.409 (3)
C4—C5	1.418 (3)	C19—H19	0.9300
C4—C9	1.418 (2)	C20—C21	1.349 (3)
C5—C6	1.343 (3)	C20—H20	0.9300
C5—H5	0.9300	C21—C22	1.406 (3)
C6—C7	1.405 (3)	C21—H21	0.9300
C6—H6	0.9300	C22—C27	1.411 (3)
C7—C8	1.361 (3)	C22—C23	1.421 (2)
C7—H7	0.9300	C23—C24	1.417 (2)
C8—C9	1.428 (2)	C24—C25	1.362 (3)
C8—C11	1.502 (2)	C24—H24	0.9300
C9—C10	1.417 (3)	C25—C26	1.398 (3)
C10—H10	0.9300	C25—H25	0.9300
C11—H11	0.9800	C26—C27	1.360 (3)
C12—C14	1.521 (2)	C26—H26	0.9300
C12—H12A	0.9700	C27—H27	0.9300
			
C17—O1—C15	110.63 (12)	C13—C14—C16	110.89 (14)
C17—O2—C16	110.43 (13)	C12—C14—C16	111.14 (13)
C12—O3—C11	111.94 (13)	C13—C14—C15	111.88 (14)
C11—O4—C13	111.16 (14)	C12—C14—C15	108.36 (13)
C10—C1—C2	120.7 (2)	C16—C14—C15	107.20 (12)
C10—C1—H1	119.6	O1—C15—C14	112.09 (13)
C2—C1—H1	119.6	O1—C15—H15A	109.2
C3—C2—C1	120.09 (19)	C14—C15—H15A	109.2
C3—C2—H2	120.0	O1—C15—H15B	109.2
C1—C2—H2	120.0	C14—C15—H15B	109.2
C2—C3—C4	120.93 (19)	H15A—C15—H15B	107.9
C2—C3—H3	119.5	O2—C16—C14	110.83 (13)
C4—C3—H3	119.5	O2—C16—H16A	109.5
C3—C4—C5	121.35 (18)	C14—C16—H16A	109.5
C3—C4—C9	119.49 (19)	O2—C16—H16B	109.5
C5—C4—C9	119.16 (17)	C14—C16—H16B	109.5
C6—C5—C4	120.62 (19)	H16A—C16—H16B	108.1
C6—C5—H5	119.7	O2—C17—O1	110.54 (11)
C4—C5—H5	119.7	O2—C17—C18	111.03 (13)
C5—C6—C7	120.7 (2)	O1—C17—C18	106.80 (12)
C5—C6—H6	119.6	O2—C17—H17	109.5
C7—C6—H6	119.6	O1—C17—H17	109.5
C8—C7—C6	121.05 (19)	C18—C17—H17	109.5
C8—C7—H7	119.5	C19—C18—C23	119.86 (16)
C6—C7—H7	119.5	C19—C18—C17	121.19 (15)
C7—C8—C9	119.73 (16)	C23—C18—C17	118.84 (14)
C7—C8—C11	120.58 (16)	C18—C19—C20	120.83 (18)
C9—C8—C11	119.59 (16)	C18—C19—H19	119.6
C10—C9—C4	117.72 (16)	C20—C19—H19	119.6
C10—C9—C8	123.65 (16)	C21—C20—C19	120.41 (19)
C4—C9—C8	118.63 (17)	C21—C20—H20	119.8
C1—C10—C9	121.03 (18)	C19—C20—H20	119.8
C1—C10—H10	119.5	C20—C21—C22	121.00 (18)
C9—C10—H10	119.5	C20—C21—H21	119.5
O4—C11—O3	110.36 (12)	C22—C21—H21	119.5
O4—C11—C8	110.41 (14)	C21—C22—C27	121.80 (19)
O3—C11—C8	106.74 (13)	C21—C22—C23	119.21 (18)
O4—C11—H11	109.8	C27—C22—C23	118.98 (19)
O3—C11—H11	109.8	C24—C23—C22	117.77 (16)
C8—C11—H11	109.8	C24—C23—C18	123.62 (15)
O3—C12—C14	111.92 (13)	C22—C23—C18	118.60 (16)
O3—C12—H12A	109.2	C25—C24—C23	121.35 (19)
C14—C12—H12A	109.2	C25—C24—H24	119.3
O3—C12—H12B	109.2	C23—C24—H24	119.3
C14—C12—H12B	109.2	C24—C25—C26	120.7 (2)
H12A—C12—H12B	107.9	C24—C25—H25	119.7
O4—C13—C14	110.57 (13)	C26—C25—H25	119.7
O4—C13—H13A	109.5	C27—C26—C25	119.58 (19)
C14—C13—H13A	109.5	C27—C26—H26	120.2
O4—C13—H13B	109.5	C25—C26—H26	120.2
C14—C13—H13B	109.5	C26—C27—C22	121.57 (19)
H13A—C13—H13B	108.1	C26—C27—H27	119.2
C13—C14—C12	107.37 (13)	C22—C27—H27	119.2
			
C10—C1—C2—C3	0.0 (3)	C17—O1—C15—C14	56.96 (17)
C1—C2—C3—C4	0.6 (3)	C13—C14—C15—O1	70.48 (17)
C2—C3—C4—C5	178.8 (2)	C12—C14—C15—O1	−171.34 (13)
C2—C3—C4—C9	−1.1 (3)	C16—C14—C15—O1	−51.30 (18)
C3—C4—C5—C6	−178.1 (2)	C17—O2—C16—C14	−59.81 (17)
C9—C4—C5—C6	1.9 (3)	C13—C14—C16—O2	−70.38 (17)
C4—C5—C6—C7	−2.7 (4)	C12—C14—C16—O2	170.25 (13)
C5—C6—C7—C8	0.7 (3)	C15—C14—C16—O2	52.01 (18)
C6—C7—C8—C9	2.1 (3)	C16—O2—C17—O1	63.95 (16)
C6—C7—C8—C11	−174.38 (18)	C16—O2—C17—C18	−177.72 (12)
C3—C4—C9—C10	1.0 (2)	C15—O1—C17—O2	−62.50 (17)
C5—C4—C9—C10	−178.95 (17)	C15—O1—C17—C18	176.62 (13)
C3—C4—C9—C8	−179.18 (15)	O2—C17—C18—C19	−9.1 (2)
C5—C4—C9—C8	0.9 (2)	O1—C17—C18—C19	111.48 (16)
C7—C8—C9—C10	177.00 (17)	O2—C17—C18—C23	174.59 (12)
C11—C8—C9—C10	−6.5 (2)	O1—C17—C18—C23	−64.84 (17)
C7—C8—C9—C4	−2.8 (2)	C23—C18—C19—C20	1.7 (2)
C11—C8—C9—C4	173.70 (14)	C17—C18—C19—C20	−174.60 (15)
C2—C1—C10—C9	−0.1 (3)	C18—C19—C20—C21	0.9 (3)
C4—C9—C10—C1	−0.4 (2)	C19—C20—C21—C22	−1.8 (3)
C8—C9—C10—C1	179.76 (17)	C20—C21—C22—C27	−178.67 (17)
C13—O4—C11—O3	62.51 (17)	C20—C21—C22—C23	0.2 (3)
C13—O4—C11—C8	−179.72 (12)	C21—C22—C23—C24	−176.99 (15)
C12—O3—C11—O4	−59.83 (17)	C27—C22—C23—C24	1.9 (2)
C12—O3—C11—C8	−179.84 (13)	C21—C22—C23—C18	2.3 (2)
C7—C8—C11—O4	−11.1 (2)	C27—C22—C23—C18	−178.80 (14)
C9—C8—C11—O4	172.40 (13)	C19—C18—C23—C24	176.01 (15)
C7—C8—C11—O3	108.84 (19)	C17—C18—C23—C24	−7.6 (2)
C9—C8—C11—O3	−67.62 (18)	C19—C18—C23—C22	−3.2 (2)
C11—O3—C12—C14	55.23 (17)	C17—C18—C23—C22	173.15 (13)
C11—O4—C13—C14	−60.70 (17)	C22—C23—C24—C25	0.0 (2)
O4—C13—C14—C12	53.24 (18)	C18—C23—C24—C25	−179.21 (16)
O4—C13—C14—C16	−68.36 (17)	C23—C24—C25—C26	−1.8 (3)
O4—C13—C14—C15	172.01 (13)	C24—C25—C26—C27	1.6 (3)
O3—C12—C14—C13	−51.13 (18)	C25—C26—C27—C22	0.4 (3)
O3—C12—C14—C16	70.32 (17)	C21—C22—C27—C26	176.72 (19)
O3—C12—C14—C15	−172.14 (13)	C23—C22—C27—C26	−2.2 (3)

### Hydrogen-bond geometry (Å, °)

**Table:**

Cg5 and Cg6 are the centroids of the C18–C23 and C22–C27 naphthyl rings, respectively.

**Table:**

*D*—H···*A*	*D*—H	H···*A*	*D*···*A*	*D*—H···*A*
C16—H16B···Cg5^i^	0.97	2.95	3.5827 (19)	124
C27—H27···Cg6^ii^	0.93	2.94	3.754 (2)	147

Symmetry codes: (i) *x*, *y*−1, *z*; (ii) −*x*, *y*+1/2, −*z*+1/2.
